# Human papillomavirus is not associated to non-small cell lung cancer: data from a prospective cross-sectional study

**DOI:** 10.1186/s13027-019-0235-8

**Published:** 2019-08-02

**Authors:** Estela Maria Silva, Vânia Sammartino Mariano, Paula Roberta Aguiar Pastrez, Miguel Cordoba Pinto, Emily Montosa Nunes, Laura Sichero, Luisa Lina Villa, Cristovam Scapulatempo-Neto, Kari Juhani Syrjanen, Adhemar Longatto-Filho

**Affiliations:** 1 Teaching and Research Institute, Molecular Oncology Research Center, Barretos Cancer Hospital – Pio XII Foundation, Barretos, Brazil; 2 Department of Chest, Barretos Cancer Hospital - Pio XII Foundation, Barretos, Brazil; 3 Center for Translational Research in Oncology, Instituto do Cancer do Estado de Sao Paulo – ICESP, São Paulo, Brazil; 4 Department of Radiology and Oncology, School of Medicine, Universidade de São Paulo, São Paulo, Brazil; 5 Associate Researcher in COI Institute, Rio de Janeiro, Brazil; 6 Department of Clinical Research - Biohit Oyj, Helsinki, Finland; 7 Medical Laboratory of Medical Investigation (LIM) 14. Department of Pathology, Faculty of Medicine, Universidade de São Paulo, São Paulo, Brazil; 8 Research Institute of Life and Health Sciences (ICVS), School of Medicine, University of Minho, Braga, Portugal; 9 ICVS, 3B’s - Associated Laboratory to the Government of Portugal, Braga, Guimarães, Portugal; 10 Surgical Sciences Research Domain Life and Health Sciences Research Institute (ICVS) School of Medicine, University of Minho, Campus de Gualtar, 4710-057 Braga, Portugal

**Keywords:** Non-small cell lung cancer, Lung neoplasms, Papillomaviridae, Papillomavirus DNA

## Abstract

**Background:**

The pathogenesis of lung cancer is triggered by a combination of genetic and environmental factors, being the tobacco smoke the most important risk factor. Nevertheless, the incidence of lung cancer in non-smokers is gradually increasing, which demands the search for different other etiological factors such as occupational exposure, previous lung disease, diet among others. In the early 80’s a theory linked specific types of human papillomavirus (HPV) to lung cancer due to morphological similarities of a subset of bronchial squamous cell carcinomas with other HPV-induced cancers. Since then, several studies revealed variable rates of HPV DNA detection. The current study aimed to provide accurate information on the prevalence of HPV DNA in lung cancer.

**Methods:**

Biopsies were collected from 77 newly diagnosed non-small cell lung cancer (NSCLC) patients treated at the Thoracic Oncology Department at Barretos Cancer Hospital. The samples were formalin fixed and paraffin embedded (FFPE), histologic analysis was performed by an experienced pathologist. DNA was extracted from FFPE material using a commercial extraction kit and HPV DNA detection was evaluated by multiplex PCR and HPV16 specific real-time PCR.

**Results:**

HPV was not identified in any of the samples analysed (69).

**Conclusions:**

Our data demonstrated a lack of HPV DNA in a series of NSCL cancers.

## Background

Lung cancer is the major cause of cancer-related death worldwide. The International Agency for Research on Cancer (IARC) estimated 1.8 million new cases for 2016 [1]. Although the pathogenesis of lung cancer is complex and arises due to a combination of genetic and environmental factors [2], tobacco smoke is consensually assumed as the strongest factor associated to this disease development, worldwide [3]. However, epidemiologic data obtained from different geographical regions have revealed that approximately 25% of all cases of lung cancer are not attributable to tobacco use [4]. Other risk factors have attracted the attention of physicians because the incidence of this disease among non-smokers has increased in the past years [5–7]. Consequently, other etiological factors have been investigated including occupational exposure, previous lung disease, diet, among others [4, 8]. Additional, human papillomavirus (HPV) infection has emerged as a potential etiological agent for certain types of bronchogenic carcinomas. Syrjänen and col. (1979) [9] suggested in the early 80’s that oncogenic HPVs could be responsible for lung cancer pathogenesis [10] due to the morphological resemblances within a subset of bronchial squamous cell carcinomas compared with the clinical manifestations of HPVs in the female genital tract [9, 11, 12]. Since then, numerous studies have pointed out an inquisitive heterogeneity on HPV DNA detection rates in lung cancers, with the highest rates detected among Asians [13–18]. European studies reported low or no HPV prevalence (0–10%) compared with the 22% (4/18) of Asian study. In the latter, HPV16 and HPV18 prevalence were 11.6 and 8.8%, respectively. However, the authors suggested more attention to study design and laboratory detection methods for analyzing this theme [16].

Syrjanen (2012) [19] performed a systematic review and formal meta-analysis of the literature reporting on HPV detection in lung cancer using MEDLINE and Current Contents platforms and found that geographical origin, histopathological types of cancer and HPV detection methods were not significant co-variates accounting for the heterogeneity of the HPV prevalence in lung cancer. Related to HPV infection as risk for lung cancer development, in 3,083 cases and 4,328 controls in two retrospective case-control studies and, one prospective nested case-control study, no association was observed between HPV infections and lung cancer development; also, viral oncogenes, HPV antibodies and DNA HPV were not related to lung cancer survival [20].

HPV infection in lung tissue was evaluated by another meta-analysis using the search terms “lung cancer”, “human papillomavirus”, “HPV” and their combinations and, antagonistically, the results suggested that HPV infection in lung tissue have a strong association with lung cancer development [21]. Another meta-analysis based on PubMed, Ovid and Web of Science to identify case-control studies and cohort studies that detected HPV in lung carcinomas included 30 or more cases published before Feb 28, 2017 and corroborated that HPV16 and HPV18 infection significantly increase the risk of lung cancer [22].

Additionally, a Chinese a study developed to identify the association between HPV positive rate and smoking in lung cancer (LC) patients observed that HPV infections are associated with smoking in LC patients and the association may relate to different regions [23]. Lung cancer study of Brazilian patients, the presence of HPV was detected by PCR followed by genotyping, found HPV in 33 of the 63 samples, and HPV types 16 and 18 were detected with frequencies of 81% (27/33) and 19% (6/33), respectively. Also, the expression of the E6 and E7 oncoproteins HPV type specific, evaluated by immunohistochemical, was detected in 28/33 samples and 25/33 samples, respectively [24].

Due to discrepancies among the results across the world and previous work from our country, we aimed to provide detailed additional data on the HPV burden in lung cancer by evaluating the prevalence of HPV DNA in samples of non-small cell lung cancer (NSCLC) using a specific method for this. Furthermore, p16 expression was accessed by immunohistochemistry (IHC) to further address if it’s immunoexpression could be considered as a surrogate marker of HPV activity in NSCLC specimens.

## Materials and methods

### Inclusion criteria

Samples were achieved from a prospective analysis of patients submitted to the bronchoscopy admitted at Barretos Cancer Hospital between 2013 and 2015. All biopsies were performed for diagnosis and all the patients (77) were naïve of treatment. Patients with NSCLC diagnosis that agreed to participate were included.

### Ethics approval and consent to participate

Written informed consent was provided by all patients and the study was approved by Barretos Cancer Hospital Ethic Committee (number 920.225).

### Patient characteristics

All 77 patients were subjected to a detailed trained interviewer assisted interview for assessment of demographic, NSCLC risk factors and previous history of HPV infection variables.

### Tumor samples

Tumor samples were obtained by endobronchial and transbronchial biopsies. Specimens were routinely processed for histological diagnosis, HPV testing and for immunohistochemical analysis of p16. Pathologic stages were determined according to the American Joint Committee on Cancer (AJCC) [25], and histological classification according to criteria of the World Health Organization (WHO) [26].

### DNA extraction

Formalin-fixed, paraffin-embedded (FFPE) tumor tissue sections, previously evaluated and delimited by an expert pathologist (CSN), were deparaffinized, and using QIAamp DNA Micro kit (QIAGEN, Hilden, Germany) DNA was extracted according to the manufacturer’s recommendations. Briefly, 4–6 10 μm FFPE sections were submitted to deparaffinization with 100% xylene, followed by washes of ethanol (100, 90, and 50%) and incubation with DNA extraction buffers overnight.

### β-Globin polymerase chain reaction (PCR)

All 77 DNA samples were quantified by NanoDrop 2000 (ThermoScientific) and subjected to β-globin gene amplification using PCO3 (ACACAACTGTGTTCACTAGC 5′-3 ‘) and PCO4 (CAACTTCATCCACGTTCACC 5’-3′) primers. The amplification of a short fragment of 110 pair base of human β-globin gene indicates the integrity of DNA for subsequent analysis. Only β-globin positive samples (62) were used for HPV analysis.

### HPV analysis

HPV DNA in lung tumors was independently assessed using two different techniques based on real-time PCR. The first one consisted of TaqMan singleplex based type-specific real-time PCR targeting the E7 region of HPV 16 type using specific TaqMan probe (5′- 6FAMCAAGCAGAACCGGACAG-MGBNFQ-3′) and primers (forward 5′-GATGAAATAGATGGTCCAGC-3′ and reverse 5′- GCTTTGTACGCACAACCGAAGC-3′) [27]. Among 77 lung samples, 62 were analyzed by this assay and the reaction mixture was prepared as follows: TaqMan Universal PCR Master Mix 1 x (Applied Biosystem, Inc., EUA), 400 nM of each primer, 200 nM TaqMan probe and DNAse/RNAse free water. PCR amplifications were carried out using 5 μL of template DNA in a final volume of 25 μL in an ABI Prism 7900HT Fast Real-Time PCR System (Thermo Fisher Scientific, USA). The amplification conditions were as follows: initial denaturation for 10 min at 95 °C, followed by 40 amplification cycles of 15 s each at 95 °C and 1 min at 60 °C (annealing-extension step). Each PCR reaction included a negative (water) and a positive control (DNA extracted from CaSki cell line). All samples and controls were tested in duplicate and were considered positive when both replicates amplified in a cycle < 38.

Additionally, 29 samples were randomly chosen for testing using a type-specific PCR bead-based multiplex genotyping assay that combine multiplex PCR and bead-based Luminex technology (Luminex Corp., Austin, TX, USA) that is able to identify 21 HPV types [28, 29], as previously described [28] and, based on available biological data [30]. Additionally, the assay has a positive control for the quality of the template DNA represented by β-globin gene primers. PCRs were performed with 10 μL of template DNA in a 96-well format in 25-μl/well final reaction volume. HPV multiplex PCR were performed using QIAGEN Multiplex PCR Kit (Qiagen, Dusseldorf, Germany), according to manufacturer’s instructions. Each reaction consisted of 45 cycles: 94 °C for 30 s, 63 °C for 3 min, and 72 °C for 90 s. The first cycle was preceded by incubation at 95 °C for 15 min and the last cycle was extended for 10 min at 72 °C. PCR negative control consisted of a reaction mix without DNA. Hybridizations were performed according to Schmitt et al. (2006) [31]. For each HPV type-specific probe, the mean fluorescence intensity (MFI) values obtained when no PCR product was added to the hybridization mixture was considered as background. The cutoffs were calculated by adding 5 MFI to 1.1 times the value of the median background. MFI values > 20 were considered positive.

### Immunohistochemistry

Sections of 4 μm containing representative tumour areas were used for IHC, which was performed using Ventana Benchmark ULTRA automated system (Ventana Medical Systems, Inc., Tucson, AZ). Antigen retrieval was performed using cell conditioning 1 buffer (CC1) at 95 °C for 64 min. For detection of immune reaction we used the ultraView Universal DAB Detection Kit polymer amplification system (Ventana Medical Systems, Mannheim, Germany) according to manufacturer’s instructions. Mouse monoclonal anti-human antibody against p16INK4A protein, Clone E6H4™, ready for use (Ventana, USA) was used as primary antibody. Samples with strong and diffuse nuclear and cytoplasmic staining in more than 70% of the cells were considered positive [25, 26]. All slides were analyzed by two observers (CSN and ALF) who revised discordant cases for achieving consensus. A cervical adenocarcinoma was used as a positive control for p16 staining and negative controls were obtained by omitting the primary antibodies [32, 33].

### Statistical analyses

Frequencies were obtained using the IBM® SPSS® Statistics 21.0 software for Windows (IBM Corporation, Somers, NY, USA).

## Results

**Fig. 1:**
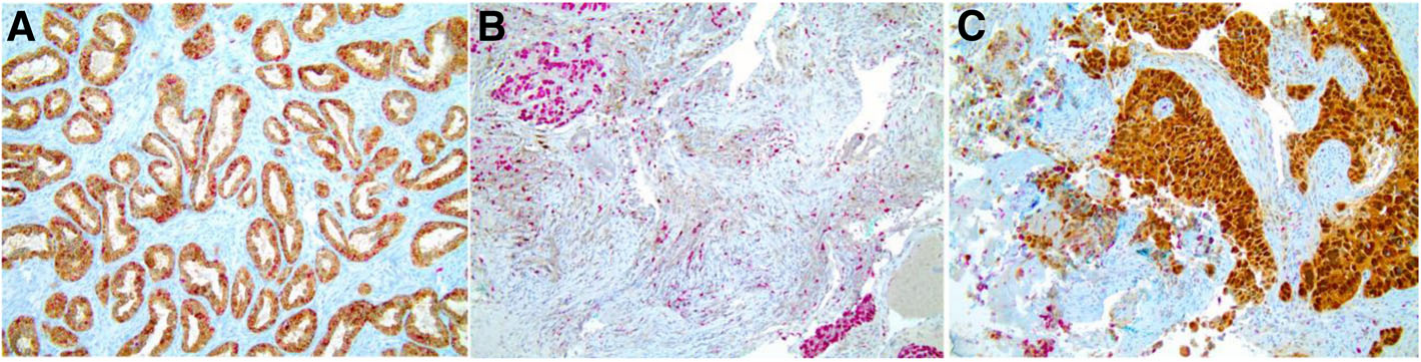
Immunohistochemical staining of tumor for p16 expression. **a** Adenocarcinoma cervical used as positive control. **b** NSCLC sample scored as negative p16 expression. **c** NSCLC sample considered as positive p16 expression. As cell proliferation nuclear marker was used Ki-67. Magnification: × 20

Among 77 samples included in this study, 15 were excluded due to the lack of β-Globin amplification and 62 were suitable for further analysis: 41 (66.1%) squamous cell carcinomas (SCC) and 21 (33.9%) adenocarcinomas. The SCC staging was as follow: 2 (3.4%) stage I, 4 (6.8%) stage II, 26 (44.1%) stage III and 27 (45.8%) stage IV. Additionally, 27 tissue samples (51.9%) were classified as well or moderately differentiated, and 25 (48.1%) as poorly differentiated. Most patients were male (*n* = 47, 75.8%), married (*n* = 40, 64.5%), and 54 (87.1%) have lived in rural area. As a consequence, 21 (33.9%) patients were farmer. Further characteristics of the patients included in this study are summarized in Table 1. The immunohistochemical staining of tumor for p16 and Ki-67 are illustrated in Figure 1.

**Table 1:** General Characteristics of NSCLC patients

Characteristic	Frequency (*n*)	Valid Percent (%)
Alcohol intake		
No	7	11.3
Yes	55	88.7
Smoking habits		
No	9	14.5
Yes	53	85.5
Exposure to pesticide and insecticides		
No	32	51.6
Yes	30	48.4
Exposure to asbestos		
No	45	80.4
Yes	11	19.6
Quantity of sexual partners^a^		
1–10	34	56.6
11–100	22	36.6
> 100	4	6.7
Oral sex^a^		
No	42	71.2
Yes	17	28.8
History of Sexually transmitted disease^a^		
No	51	83.6
Yes	10	16.4
History of Genital warts^a^		
No	57	93.4
Yes	4	6.6
Total	62	100

^a^Missing data

None of the 62 NSCLC samples tested was positive for HPV using any of the techniques. Furthermore, p16 immunoreaction of all NSCLC was analyzed and it was observed positive expression in 10 (14,3%) cases. The analysis of positive cases for the expression of p16 (10/70) in relation to socio-demographic information, lifestyle and clinical-pathological findings showed no statistically significant correlation (Table 2).

**Table 2:** Characterization of the case group in relation to the expression of p16 and socio-demographic variables, lifestyle and clinical data. Cancer Hospital of Barretos, January/2013 to October/2015

Variable	Category	p16 expression				p
		Positive		Negative		
		n (*)	(%)	n (*)	(%)	
Gender	Female	4	(40,0)	13	(21,7)	0,242
	Male	6	(60,0)	47	(78,3)	
Race	Não Branco	3	(30,0)	12	(20,3)	0,679
	Branco	7	(70,0)	47	(79,7)	
Alcohol intake	Yes	8	(80,0)	53	(88,3)	0,607
	No	2	(20,0)	7	(11,7)	
Smoking habits	Yes	5	(50,0)	36	(60,0)	0,731
	No	5	(50,0)	24	(40,0)	
Histological types	Squamous cell carcinoma	6	(60,0)	37	(61,7)	0,999
	Adenocarcinoma	4	(40,0)	23	(38,3)	
Differentiation Degree	Well or Moderate	3	(30,0)	25	(52,1)	0,301
	Poor	7	(70,0)	23	(47,9)	
T	T1 – T2	2	(20,0)	12	(20,7)	0,999
	T3 – T4	8	(80,0)	46	(79,3)	
N	N0	1	(10,0)	10	(17,2)	0,999
	N positive	9	(90,0)	48	(82,8)	
M	M0	4	(40,0)	28	(48,3)	0,739
	M1	6	(60,0)	30	(51,7)	
Stage	I – II	0	(0,0)	6	(10,5)	0,580
	III – IV	10	(100,0)	51	(89,5)	
Total		10	(100)	60	(100)	

(*) Cases with missing values were excluded from the analysis

*n* number; TNM staging: a system based on the size and/ or the extension of the primary tumor (T), the number of compromised lymph nodes (N) and the presence of metastasis (M). Fisher’s exact test was used. It was considered statistically significant *p* < 0.05

## Discussion

The main goal of this study was to evaluate the presence of HPV DNA in NSCLC. Many contradictory results have been found among the reports published about this issue; the majority of discordances may likely be attributable to the differences in methodology used to identify HPV and the fact that many studies have no control regarding the quality of the samples preservation retrospectively selected for analysis. After all, it is not a surprise that an assertive attention about the real meaning of HPV in lung cancer is currently questionable. We did not find any NSCLC positive sample for HPV DNA among the samples we evaluated, despite the use of a very sensitive methodology we used to identify HPV, and the stringent conditions to preserve the samples prospectively collected, including rigorous control of quality in each step of the study. This final result is in agreement with some results obtained worldwide from different studies groups [34, 35]. Ywakawa et al. [36], e.g., analysed HPV-16, 18 and 33 DNA in 275 lung adenocarcinoma samples using 2 different methodologies (PCR multiplex and nested PCR) and also found no positive samples. Additionally, in another series [37] comprising 196 samples (100 adenocarcinoma and 96 squamous cell carcinoma), using in situ hybridization (ISH) capable of detecting high-risk HPV DNA (16, 18, 31, 33, 35, 39, 45, 51, 52, 56, 58, 68 and 70) no HPV DNA was observed. These data argue against the possible participation of HPV in NSCLC carcinogenesis [36, 37].

On the other hand, some studies have reported correlation between HPV and lung cancer [21]. Syrjänen et al. [38] detected HPV DNA in 4/77 (5.2%) NSCLC samples, three of which were positive for HPV-16 and one sample for HPV -6 / -16 coinfection, using multiplex PCR (Luminex®). Sarchianaki et al. [2] analysed 100 samples of NSCLC using real-time PCR methodology (GP5 + / GP6 +) and, 19 (19%) samples tested positive for HPV DNA. In order to genotype the positive samples, LINEAR ARRAY HPV Genotyping (Roche) capable of detecting 37 high and low risk HPV genotypes was also used, and 42.1% (8/19) of the positive samples were HPV-16 [2]. Moreover, Yu et al. [39] analysed 261 samples (107 squamous cell carcinoma, 63 adenocarcinoma and 91 non-tumour samples as control) by real-time PCR and INNO LIPA; 59.8% (64/107) in SCC, 17.5% (11/63) of the adenocarcinoma samples and 23.1% in the control samples were positive for HPV DNA. HPV-16 and / or 18 were found in 79.7% in SCC, 72.7% in adenocarcinoma and 14.3% in the control samples [39]. Together, these data indicate a wide rate of variation in the frequency of HPV among lung cancers with the highest frequency of virus being reported in East Asian countries, with a prevalence variation of 11.8–55.0% [16]. Finally, in a recent meta-analysis [40], 46 studies that demonstrated a higher prevalence of HPV in Asian countries (28.1%) when compared with European countries (8.4%) and countries from North and South America (21.3%), with regional differences between countries being observed. When the analysis was limited to HPV-16 and 18, which are the HPV types of higher oncogenic risk, a significant higher prevalence was observed in Asia (23.1%) in relation to Europe (4.4%) or the Americas (15.6%). This is interesting because some studies suggest that the heterogeneity in the prevalence of HPV in lung cancer is mainly due to the geographic differences, the different histological types analysed and the different detection methods used [19], besides the sample size, demographic composition of each study and host-specific factors [14, 16]. There is a consensus among most of the studies that it is necessary to investigate more cases to understand the real role (if any) of HPV in pulmonary carcinogenesis [14, 16, 20, 37, 40].

Still in an attempt to explore the relationship of HPV with NSCLC, IHC was performed in NSCLC paraffin samples to evaluate the expression of p16, a protein considered an indirect marker for HPV infection, since in SCC and adenocarcinoma of uterine cervix and, in a fraction of oropharyngeal squamous cell carcinomas, the overexpression of p16 is strongly related to HPV infection [37]. However, the relationship between p16 expression and NSCLC is not well established [41] and our data were not able to demonstrate the presumed relationship. Chang et al. [37], in addition to the evaluating the presence of HPV DNA, also evaluated the expression of p16 on NSCLC by IHC, and as a result, no correlation between the HPV and p16 expression was determined. Other authors, such as van Boerdonk et al. [42], Bishop et al. [43] and Doxtader et al. [41], presented similar results, with 20 to 30% of NSCLC, both adenocarcinoma and squamous cell carcinoma, positive for p16 and negative for HPV DNA. In addition, these authors concluded that IHQ for p16 cannot be considered a surrogate method for assessing the presence of HPV in lung cancer.

## Conclusion

Our data shows lack of HPV infection in a series of NSCL cancers. The few p16-positive cases are therefore unrelated to HPV. Several reports evaluating the prevalence of HPV DNA are increasing and new evidences with the HPV infection and NSCL coexistence are emerging [40–43]. However, there is still a lack of robust evidences of the possible participation of HPV in NSCLC carcinogenesis [44].

## Data Availability

All data generated or analyzed during this study are included in this manuscript.
